# Erosive pustular dermatosis after herpes zoster

**DOI:** 10.1002/hsr2.277

**Published:** 2021-05-05

**Authors:** Hüsna Güder, Semih Güder, Şükrü Yıldırım

**Affiliations:** ^1^ Department of Dermatology, Medical Faculty Maltepe University Istanbul Turkey; ^2^ Department of Dermatology, Medical Faculty Bezmialem Vakif University Istanbul Turkey; ^3^ Department of Pathology, Medical Faculty Maltepe University Istanbul Turkey

## 1 INTRODUCTION

Erosive pustular dermatosis of the scalp (EPDS) is an inflammatory dermatosis that shows nonmicrobial pustules, crusting, erosions, and causes scarred alopecia. It often affects the chronically photo‐damaged skin of the elderly. EPDS can be clinically confused with bacterial or fungal infections, pemphigus vulgaris, squamous cell carcinoma, and artifact dermatitis. Histopathology is not specific to the disease. Topical and systemic steroids and estrogen, topical tacrolimus, photodynamic therapies, systemic acitretin are also successful treatments.^1^, ^2^, ^3^, ^4^, ^5^ As far as we know, our case is the fourth case reported after herpes zoster so far.^2^

## 2 REPORT

**FIGURE 1:**
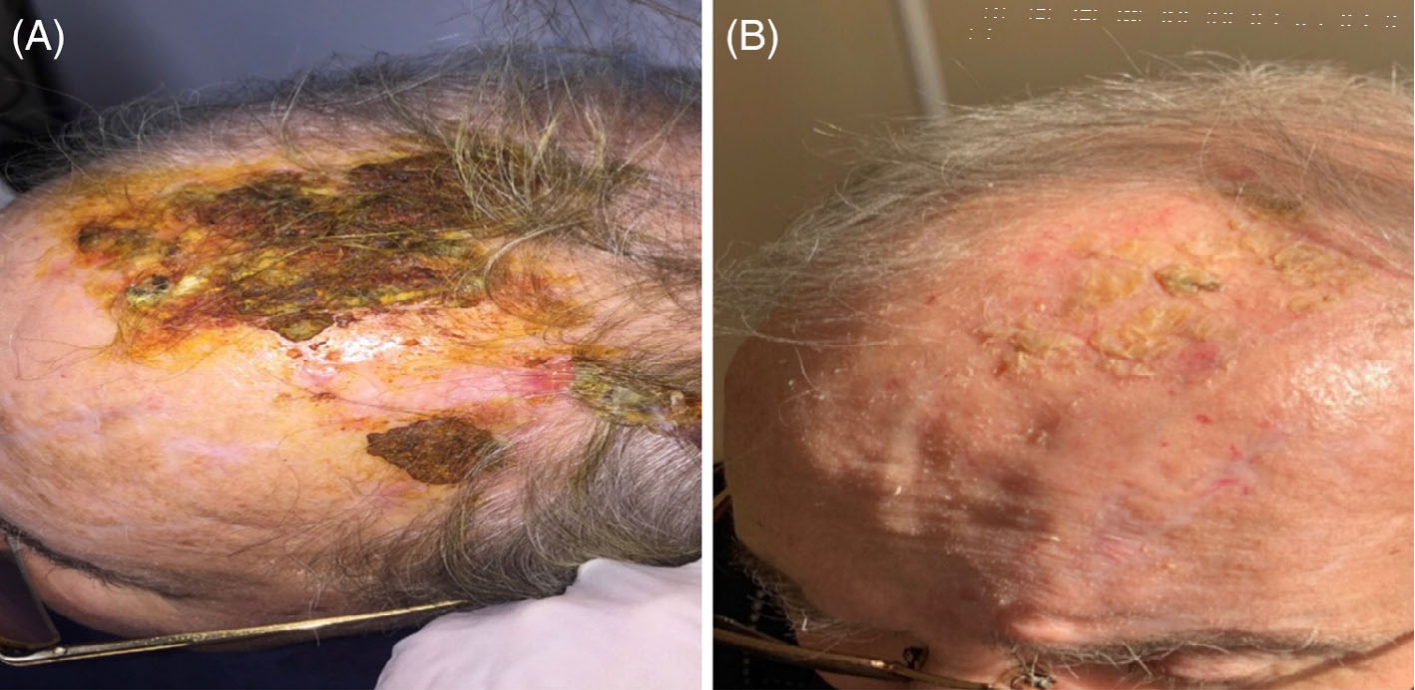
Clinic image of lesion. A, Crusty wound on the left side of his head (at admission). B, Post‐treatment appearance of the lesional site

**FIGURE 2:**
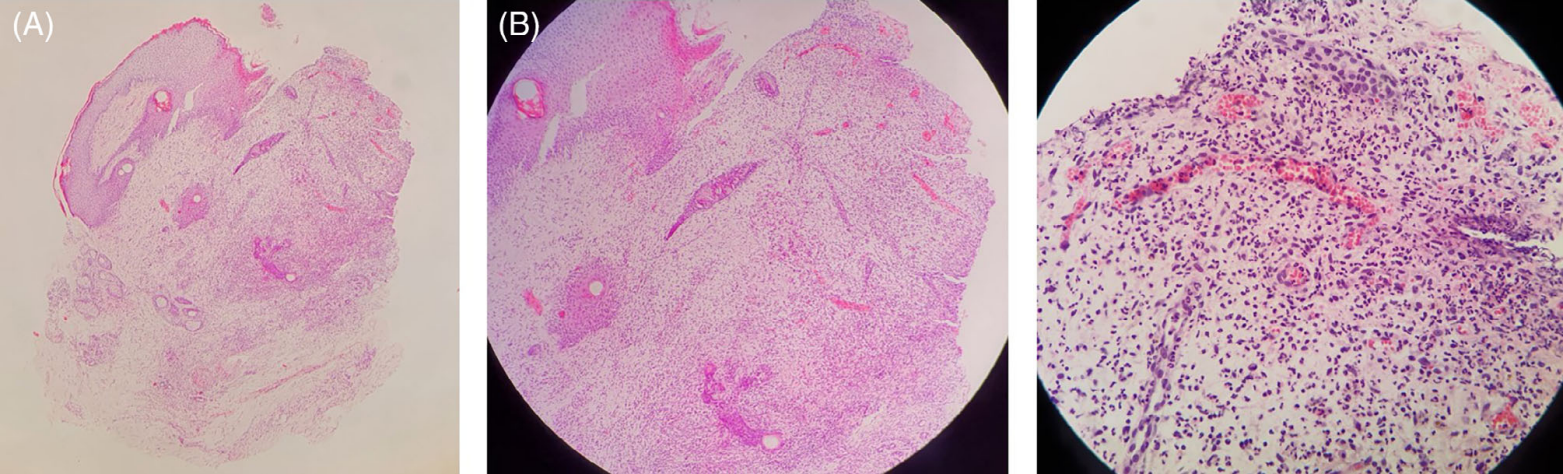
Histopathology of lesion. A, Erosion in the epidermis (×40, haematoxylin eosin). B, Perivascular and perifollicular dense neutrophilic infiltration in the dermis (×100, haematoxylin eosin). C, Vascular proliferation and fibrin structures in the vascular lumens (×400, haematoxylin eosin)

An 82‐year‐old male patient presented with a crusty wound on the left side of his head, which started 4 years ago shortly after herpes zoster infection in the same region and slowly progressed in the frontal scalp region (Figure 1A). He had type 2 diabetes mellitus and hypothyroidism. Histopathological examination revealed erosion in the epidermis, perivascular, and perifollicular dense neutrophilic infiltration in the dermis, vascular proliferation, and fibrin structures in the vascular lumens (Figure 2). It was evaluated as compatible with erosive pustular dermatosis and clobetasol propionate cream treatment was started. Within a month, the lesions healed almost completely (Figure 1B). The treatment was planned to be continued 2 days a week for 3 months. Informed consent of the patient was obtained.

## 3 DISCUSSION

Erosive pustular dermatosis is not easy to diagnose unless it is thought of. It is crucial to separate from malignancies, as it is often seen in older people and sun‐damaged skin. They can also be together. Alternatively, topical tacrolimus is used. It does not cause skin atrophy, it seems an advantage, but the possibility of causing an increased risk of skin cancer is a disadvantage.^1^, ^2^

## 4 CONCLUSION

Erosive pustular dermatosis should be considered in chronic nonhealing wounds of the scalp in the elderly.

## FUNDING

The authors have received no financial support for this article.

## CONFLICT OF INTEREST

The authors declare no conflicts of interest.

## AUTHOR CONTRIBUTIONS

Conceptualization: Hüsna Güder

Formal analysis: Semih Güder

Supervision: Şükrü Yıldırım

Writing ‐ original draft preparation: Semih Güder

Writing ‐ review and editing: Hüsna Güder

      All authors have read and approved the final version of the manuscript.

      Semih Güder had full access to all of the data in this study and takes complete responsibility for the integrity of the data and the accuracy of the data analysis.

## TRANSPARENCY STATEMENT

The lead author (Hüsna Güder), affirms that this manuscript is an honest, accurate, and transparent account of the study being reported that no important aspects of the study have been omitted; and that any discrepancies from the study as planned (and, if relevant, registered) have been explained.

## Data Availability

Data sharing is not applicable to this article as no new data were created or analyzed in this clinical case report.
